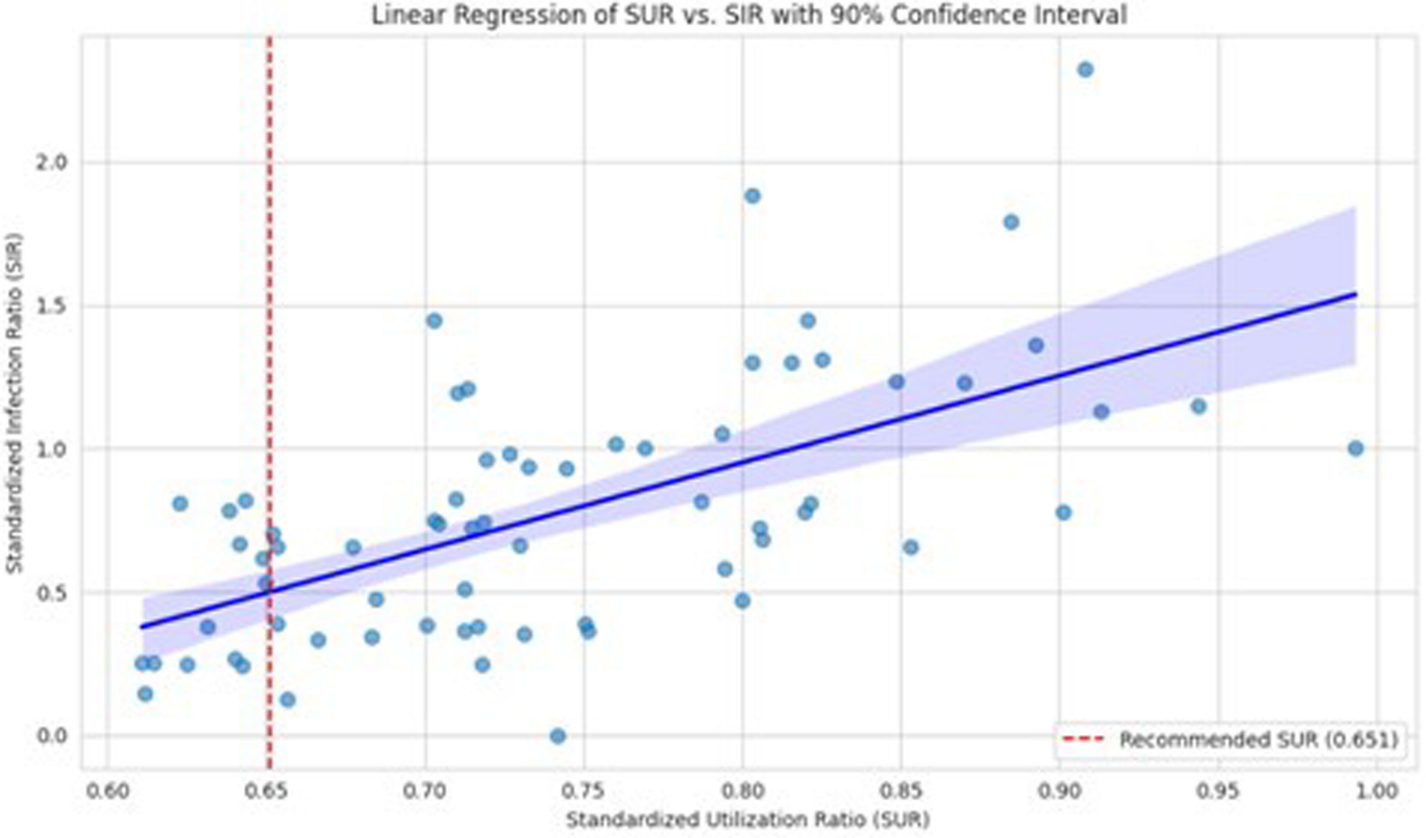# 156 Silent Gaps: Pathogen-Specific Non-Response Patterns in Occupational Exposure Management

**DOI:** 10.1017/ash.2026.10557

**Published:** 2026-06-23

**Authors:** Kathleen McMullen, Mike Joyce, Andy Hammes, David Tannehill

**Affiliations:** 1 Mercy

## Abstract

**Background:** One intervention to decrease central line associated bloodstream infections (CLABSI) standardized utilization ratios (SIR) is to decrease use of central lines. Central line use can be measured using the standardized utilization ratio (SUR), calculated by the National Healthcare Safety Network (NHSN). Unlike the SIR, decreasing the SUR all the way to zero is not an achievable goal; some patients need to have a central line for their treatment. The objective of this project was to identify a target SUR value to help reach SIR goals. **Methods:** A midwestern healthcare system set their CLABSI SIR goal at 0.5. Using paired SUR and SIR data from 6 large hospitals from July 2019 through February 2025, six machine learning methods (4 parametric, 2 non-parametric) were applied to determine the SUR required to achieve the goal SIR. The six individual model–recommended SUR values were combined using bootstrap resampling: drawing 1,000 samples (with replacement) from the six SUR estimates, computing the mean each time to produce an ensemble recommendation, and then taking the 5th and 95th percentiles of those means as a 90% confidence interval. **Results:** Over the study timeframe, the aggregated system SUR was 0.742 and aggregated SIR was 0.774 . The comprehensive analysis recommended that the system adopt an SUR target of 0.650 (90% Confidence Interval: 0.645–0.655) to achieve the central line SIR goal (Figure 1). **Conclusion:** The analysis allowed the healthcare system to set a personalized goal, using data from the member hospitals, for SUR performance. This allowed identification of hospitals that needed to focus on further interventions to decrease central line use, versus those that should pivot their CLABSI prevention interventions to other focuses.

**Figure:**